# Global patterns of raptor distribution and protected areas optimal selection to reduce the extinction crises

**DOI:** 10.1073/pnas.2018203118

**Published:** 2021-08-30

**Authors:** Carlos Cruz, Giulia Santulli-Sanzo, Gerardo Ceballos

**Affiliations:** ^a^Instituto de Ecología, Universidad Nacional Autónoma de México, Ciudad de México 04510, México

**Keywords:** raptor conservation, conservation prioritization, extinction crises, avian predators, protected areas

## Abstract

Globally, human-caused environmental impacts, such as habitat loss, have seriously impacted raptor species, with some 50% of species having decreasing populations. We analyzed global patterns of distribution of all 557 raptor species, focusing on richness, endemism, geographic range, conservation status, and population trends. Highest species diversity, endemism, species at risk, or restricted species were concentrated in different regions. Patterns of species distribution greatly differed between nocturnal and diurnal species. To test the efficiency of the global protected areas in conserving raptors, we simulated and compared global reserve systems created with strategies aiming at: 1) constraining the existing system into the final solution; and 2) minimizing the socioeconomic cost of reserve selection. We analyzed three targets of species distribution to be protected (10, 20, 30%). The first strategy was more efficient in meeting targets and less efficient in cost and compactness of reserves. Focusing on actions in the existing protected areas is fundamental to consolidate conservation, and politically and economically more viable than creating new reserves. However, creating new reserves is essential to protect more populations throughout the species’ geographic range. Our findings provide a fundamental understanding of reserves to maintain raptor diversity and reduce the global population and species extinction crisis.

## Significance

Current extinction rates are caused by human activities, including habitat destruction. Here we analyze the global patterns of raptor distribution to determine priority areas for conservation. Raptors are top predators that can be used as umbrella species to help the conservation of other species. Our results provide insights into global strategies for conservation of different proportions of the geographic range of raptor species, minimizing socioeconomic conflict. These findings are fundamental to guide conservation actions that may help avert the massive current extinction crises.

Human activities are responsible for the catastrophic decline and extinction of thousands of animal and plant species throughout the world, and this loss is occurring at unprecedented rates (1–6). Raptors are some of the most threatened vertebrate taxa, and in the last three decades many species have experienced severe population declines or faced extinction (7, 8). This threat is primarily the result of habitat loss and fragmentation, pollution, human–wildlife conflicts, and global climate alterations (5–9). The relationship between raptors and humans is seemingly contradictory. Historically, raptors were important icons in different cultures and have been used for falconry in many places globally. In contrast, they have been persecuted due to conflicts with human interests, namely predation of game species and livestock (9). As top predators, raptors are flagship, umbrella, or keystone species and are used as surrogate species in biodiversity conservation efforts (9, 10). From an ecological perspective, raptors are top predators and scavengers, critical for maintaining ecosystem structure and function and ecosystem services (11–18). Their effect on trophic webs extends to the lower levels (e.g., herbivores), linking ecosystem processes and energy fluxes (12, 19). Raptors also indirectly increase seed production and control pest species by preying on a wide range of vertebrates and facilitate long-distance seed dispersal (19, 20).

**Fig. 1.**
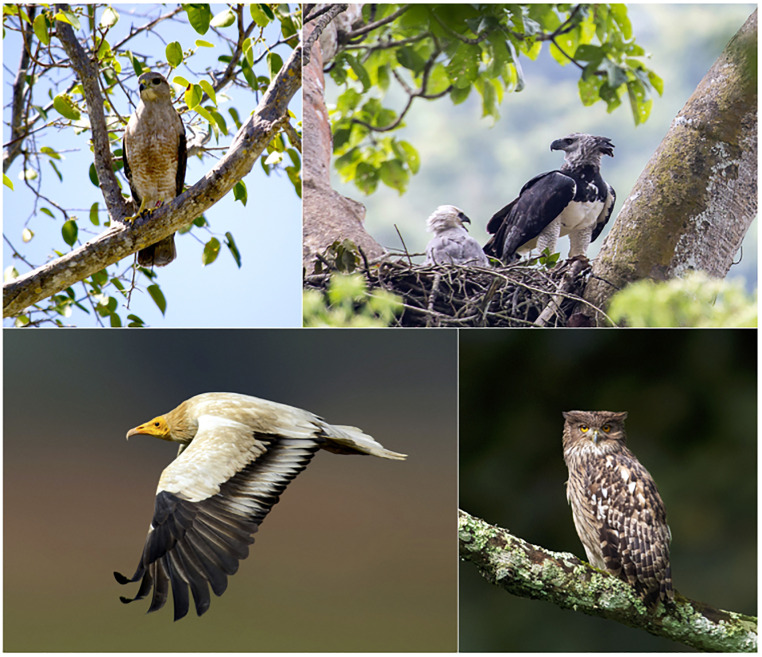
Examples of diurnal and nocturnal raptor species. Ridgway’s hawk (*Buteo ridgwayi*) is critically endangered and endemic to a very restricted region in the Dominican Republic (*Top Left* image credit: C.C.). The Harpy eagle (*Harpia harpyja*) is the largest eagle of the Neotropical region, with decreasing populations (*Top right* image credit; Carlos Navarro [photographer]). The Egyptian vulture (*Neophron percnopterus*), although widely distributed in southern Europe and northern Africa to India, is endangered (*Bottom Left* image credit: Subramaya Chandrashekar [photographer]). The brown fish owl (*Ketupa zeylonensis*) is a common species found in tropical regions of the Indian subcontinent (*Bottom Right* image credit: Subramaya Chandrashekar [photographer]).

The population decline of some raptor species during the last few decades has been so dramatic that they face extinction unless effective conservation measures are implemented (Fig. 1) (7, 8). For example, the population of the Philippine eagle (*Pithecophaga jefferyi*), the largest eagle in the world, is decreasing very rapidly due to extensive deforestation (21). Some vulture species in Asia and Africa have undergone startling population decreases in recent years because of toxification and habitat loss (22–24). As scavengers, vultures contribute to regulating ecosystem services by recycling dead matter and preventing the spread of diseases (24). Despite such beneficial roles for humans, some vulture populations have declined by over 95% in many Asian countries, such as India, because of the widespread use of diclofenac, a nonsteroidal antiinflammatory drug (22, 23). In Africa, particularly West Africa, vulture populations have decreased by an average of 95% in rural areas over the last 30 y as the result of shooting, harassment, and poisoning through feeding on carcasses of livestock treated with diclofenac (23). The Annobon scops-owl (*Otus feae*), with an estimated population of fewer than 250 individuals and restricted to Annobon Island off West Africa, was recently classified as critically endangered because of rapid habitat loss and degradation (25).

Recent assessments maintain that the world has entered the sixth mass extinction period, and habitat loss and degradation, primarily the result of rapid human population growth and its associated impacts, suggest that the future of wildlife in general, and of raptors in particular, is not encouraging (4, 6, 26). Estimates suggest that the overall vertebrate populations have decreased by 70% since 1970 (27). Evaluation of distribution patterns of taxonomic groups at different spatial scales is essential to understanding the full scope of the threats to biodiversity and developing conservation actions to mitigate them (e.g., refs. 5 and 28). Resource planners and managers can capitalize on the large amount of high-resolution global data on species distribution and conservation status to develop broad-scale strategies (1, 29–32). So, understanding the patterns of distribution of groups of species, especially those that are endangered with extinction, is fundamental to define large-scale conservation strategies (7, 8, 30–34). Two recent seminal studies have addressed the broad-scale patterns of raptor distribution, emphasizing the importance of hotspots of diversity concentrations and defining priority conservation actions, on the one hand, and the importance of supporting conservation strategies based on endemic and endangered species, on the other. Those studies have been fundamental to frame our current study (7, 8).

Increasing accessibility to global-level data on land-use and socioeconomic features provides essential tools for the valuation of potential conflicts with human activities and cost estimations (actual or relative) of conservation priority areas for specific taxa (35–38). Integrating biological and socioeconomic objectives in global conservation planning has become essential in optimizing both the selection of protected areas and investment allocation (35), especially for those countries where socioeconomic instability corresponds to poor conservation outcomes (36, 39). In this study, we present a global analysis of the distribution patterns of 557 of all raptor species in order to evaluate conservation priorities based on four parameters: 1) global distribution patterns of total, diurnal, and nocturnal species, species at risk, endemic species, and species with restricted ranges; 2) species population trends (i.e., decreasing, stable, or increasing); 3) selection of areas that minimize conflict with human activities; and 4) effectiveness of the protected area network.

## Results

### Species Diversity, Endemism, and Range Size.

There are around 557 raptor species, representing 5% of all wild birds (Fig. 1 and Table 1). More species are diurnal (321 spp, 58%) than nocturnal (236, 42%) species, varying in size from around 40 g in the black-thighed falconet (*Microhierax fringillarius*) to about 15 kg in the largest species, the Andean condor (*Vultur gryphus*). Regarding their conservation status, 166 (30%) species are either critically endangered (18 spp), endangered (25), vulnerable (57), or near-threatened (66) (Table 1).

**Table 1.** Species richness, distribution, conservation status, and number of species with decreasing populations of the raptors of the world

	Raptors of the world							
	Richness	Distribution		IUCN				
	Total no. of species	Politically endemic species	Restricted distribution (>50,000 km^2^)	Critically endangered	Endangered	Vulnerable	Near-threatened	Decreasing
All species	557	158	147	18	25	57	66	279
Diurnal species	321	61	48	11	14	32	42	172
Nocturnal species	236	94	99	7	11	25	26	107

**Fig. 2.**
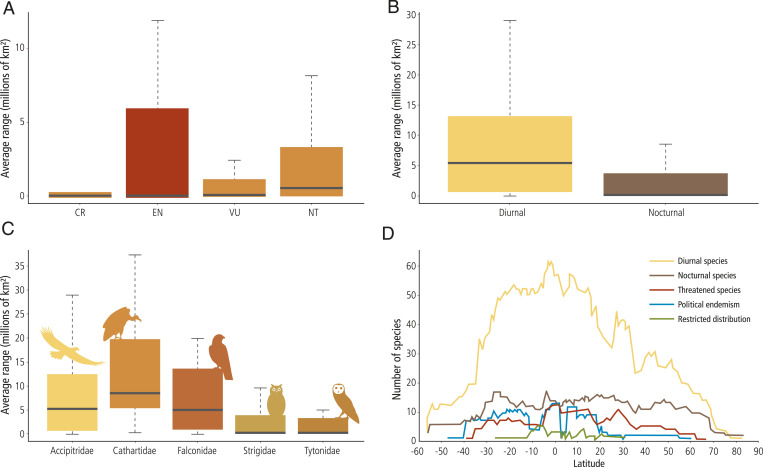
Global relationship between the average extent of the range of species and the conservation elements considered in this study: (*A*) conservation status, (*B*) activity pattern (diurnal or nocturnal), (*C*) taxonomic family (the two monospecific families, Sagittaridae and Pandionidae, were excluded), and (*D*) species richness factors of importance, including diurnal, nocturnal, conservation status, national endemism, and restricted range species. Latitude of the distribution range was also considered in the analysis.

The geographic range varied notably among species. The maximum range of 195 million km^2^ for the peregrine falcon (*Falco peregrinus*) contrasted with the smallest range of only 16 km^2^ for the Annobon scops-owl (*O. feae*). The family Cathartidae had the largest average range and the family Tytonidae had the smallest, although both the monospecific families Sagittaridae and Pandionidae had a larger range compared with that of the other five raptor families. Diurnal species had significantly larger geographic ranges than nocturnal species (Fig. 2). Some 158 (28% of the raptor total) species were politically endemic (sensu 4), meaning that they were only found in a single country. Of those species, more were nocturnal than diurnal (Table 1). Critically endangered raptor species had, on average, smaller range sizes than other raptors, while endangered species generally had the largest variability (in terms of interquartile range; Fig. 2).

### Global Patterns of Raptor Distribution.

**Fig. 3.**
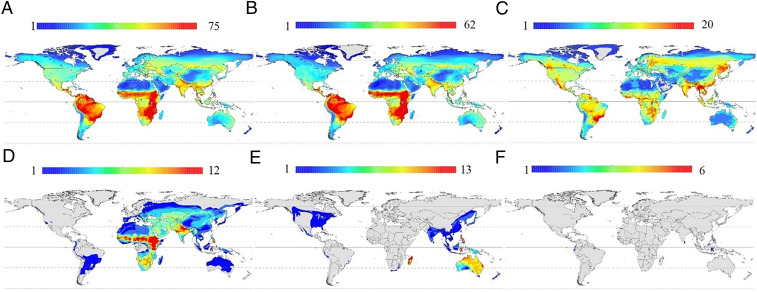
Global patterns of raptor species distribution. Species richness is shown for (*A*) all species, (*B*) diurnal species, (*C*) nocturnal species, (*D*) threatened species (IUCN categories: CR, EN, VU), (*E*) political endemism, and (*F*) restricted range species.

The global species distribution patterns, which identified biogeographic trends and revealed stark differences between diurnal and nocturnal raptors, have strong implications for conservation. Raptor species richness was unevenly distributed around the globe, varying from 1 to 65 species per 10,000-km^2^ grid cell (Fig. 3*A*). The largest number of diurnal raptor species co-occurring in a 10,000-km^2^ cell was 62 and the maximum number of nocturnal species in this same areal extent was 20. The highest diversity occurred in the South American Andes, Himalayan and Indo-Malayan regions, and some Pacific islands (Fig. 3*B*). The lowest numbers of species were in the polar regions and the arid and temperate zones in both hemispheres. The geographic patterns of species richness differed greatly between diurnal and nocturnal species. In general, the nocturnal raptor species, in contrast to diurnal ones, were more widespread globally, had a lower diversity per cell area, and were more frequently found at higher latitudes (Fig. 3*C*). South America and Sub-Saharan Africa, except the Congo Basin, had the highest species richness of diurnal raptors. At a country level, Indonesia had the largest number of total raptor species (116 spp, 21% of the total), followed by Colombia (103, 18%) and Ecuador and Peru (102, 18%, each). Colombia had the highest number of diurnal species (76, 24%) and Indonesia had the largest number of nocturnal species (52, 22%). Around 40% of nocturnal and 19% of diurnal raptor species were endemic to a single country. Indonesia, the Philippines, and Madagascar had the largest number of endemic species (43, 24, and 13, respectively). Regarding species with restricted distributions, nocturnal raptors had the largest proportion of species with restricted distribution (42%) in contrast to 15% of diurnal species. Indonesia had the largest number of species with restricted distributions (29), followed by Papua New Guinea (13) and the Philippines (12).

Altogether, 37% of the raptor species were considered at risk for extinction (i.e., International Union for Conservation of Nature [IUCN]: critically endangered, endangered, or vulnerable categories), including 18% of diurnal and 19% of nocturnal species. Indonesia, Tanzania, Sudan, and Kenya had the largest number of threatened species. Of the species classified as threatened, 54% of diurnal and 47% of nocturnal species had decreasing populations, and only 14 and 3%, respectively, had increasing populations.

### Conservation Prioritization.

**Fig. 4.**
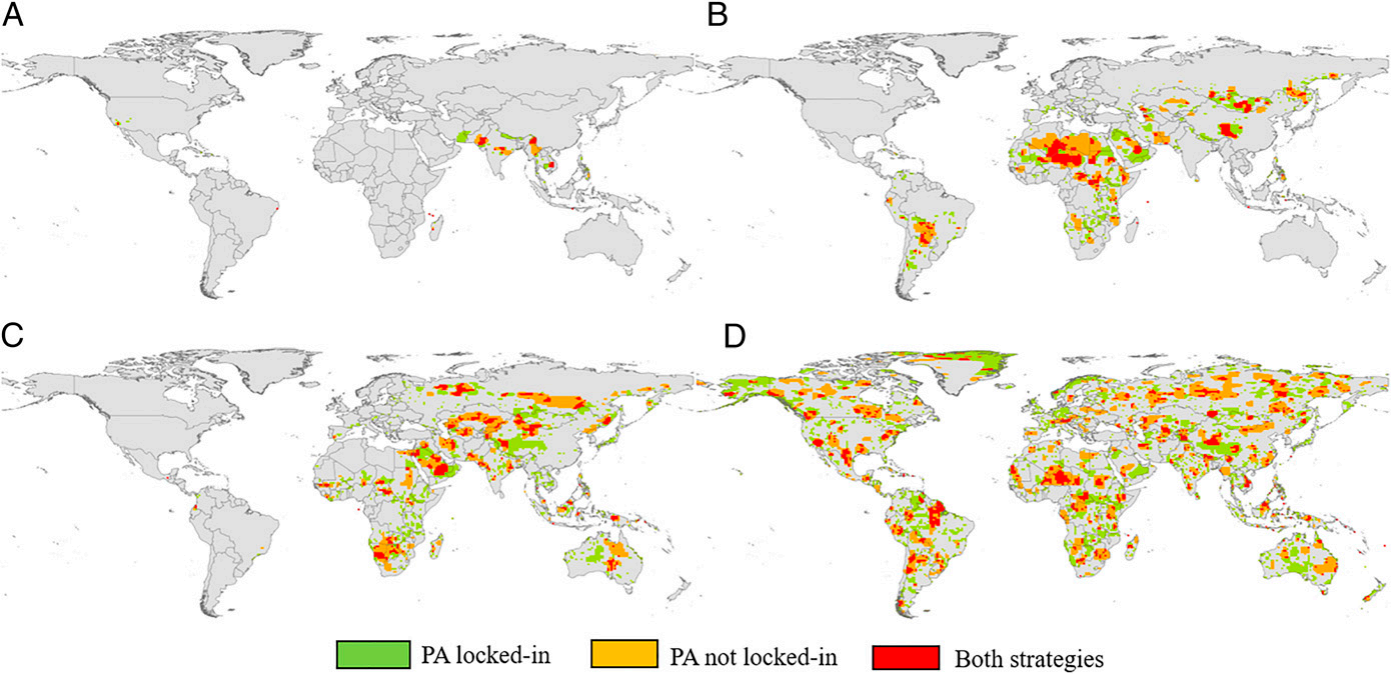
Reserve networks (Marxan solutions) for the conservation of 20% of the area of distribution of (*A*) critically endangered, (*B*) endangered, (*C*) vulnerable, and (*D*) nonthreatened species. In green is the area selected by the PA locked-in scenario; in yellow is the area selected by the PA not locked-in scenario; and in red is the overlapping area of the two solutions (*Materials and Methods*).

We used the Marxan algorithm to identify hotspots for raptor conservation at the global level, building solutions for threatened and nonthreatened species and proposing different targets of areas to be protected. Moreover, we compared two strategies of selecting reserve systems for threatened raptor conservation: In the “PA locked-in” strategy the reserve system was created around the existing protected areas (PAs), and in the “PA not locked-in” strategy the Marxan algorithm designed reserves only on the basis of species distribution, while both strategies aimed at achieving the set targets of conservation for the smallest possible cost (Fig. 4). Results of the 24 solutions created are reported in Table 2.

**Table 2.** Results of the 24 Marxan solutions for reserve networks for four conservation statuses, two strategies, and three targets of area of distribution (10, 20, 30%)

Conservation status	Strategy	Target, %	Score	Cost	Area, no. PUs	Connectivity, m	No. missing targets	Overlay strategies of same target, %	PUs in the existing PA system, %
Critically endangered	PA locked-in	10	3506.9	404.2	104.2	33850.1	0.78	18.8	69.9
		20	4179.8	663.8	170.1	41524.8	0.11	36.8	41.9
		30	4921.1	992.0	235.0	46592.4	0.38	51.9	30.6
	PA not locked-in	10	1975.7	369.3	100.9	15223.2	4.23	18.8	9.0
		20	3004.5	726.8	167.2	21677.5	4.57	36.8	12.6
		30	3956.2	1143.6	239.1	26804.4	5.80	51.9	7.5
Endangered	PA locked-in	10	24624.3	4284.2	1846.0	203400.7	0.00	6.8	83.9
		20	27438.8	4975.5	2208.9	224631.4	0.15	19.4	50.0
		30	32160.2	6420.8	2942.4	257392.3	0.45	36.2	34.1
	PA not locked-in	10	9359.6	1317.5	857.2	79055.4	5.54	6.8	10.7
		20	15549.9	2777.2	1665.7	126359.4	6.28	19.4	11.1
		30	19990.5	4429.0	2458.0	154114.0	7.60	36.2	10.6
Vulnerable	PA locked-in	10	34624.4	6097.9	2984.3	285262.3	0.07	14.2	45.6
		20	39401.5	7423.1	3480.3	319776.0	0.35	17.2	38.3
		30	45740.8	9636.7	4281.5	361039.3	0.28	29.8	30.1
	PA not locked-in	10	13697.3	2149.2	1203.2	113835.6	10.23	14.2	15.6
		20	22550.4	4459.9	2282.3	178690.1	13.46	17.2	11.8
		30	29240.9	7047.6	3310.9	220080.7	15.25	29.8	12.5
Nonthreatened	PA locked-in	10	111358.4	18641.3	11356.2	927171.0	0.02	8.6	58.2
		20	120827.8	21401.4	12126.9	994263.5	0.10	13.7	56.9
		30	132018.0	25631.2	13279.4	1063867.5	0.11	24.2	49.6
	PA not locked-in	10	37992.6	6295.9	3169.8	314500.5	20.84	8.6	15.0
		20	62428.4	12706.4	6146.5	494547.7	20.50	13.7	16.3
		30	81150.4	19516.3	9084.8	614215.5	21.18	24.2	18.4

Marxan solutions for reserve networks for conservation status, strategies, and targets of area of distribution (10%, 20%, 30%) for the raptors of the world. Score, result of the Marxan objective function; cost, cost of the solution based on the index of socioeconomic cost (*Materials and Methods*); area, number of planning units of 100-km resolution included in the final solution; connectivity, length of boundaries of every contiguous site; no. missing targets, number of species whose target was not met; overlay strategies of same target, the overlap of areas selected by the two strategies for the same target of conservation species distribution (10%, 20% and 30% of species range); PUs in the existing PA system, percentage of the total of planning units in a solution that intersects with the existing PA system. PU, planning unit.

We found that the PA locked-in strategy had higher scores, costs, and number of planning units in all the scenarios (conservation status and target of area to be protected; Table 2), and hence a lower efficiency. Even if the same cost threshold was set for both strategies, in the PA locked-in scenario the Marxan algorithm exceed the threshold to find efficient solutions. On the other hand, this strategy showed invariably higher completeness (number of species not meeting the conservation target < 1) and a significantly higher proportion of the solution already protected (minimum 30.1%, maximum 69.9%) compared with the PA not locked-in strategy (minimum 7.5%, maximum 15.6%). For the same cost threshold, the PA not locked-in strategy showed higher efficiency (lower cost and planning unit number) but failed in meeting conservation targets for all species because cost and compactness seemed to have a greater weight in the Marxan objective function in designing reserve network solutions.

**Fig. 5.**
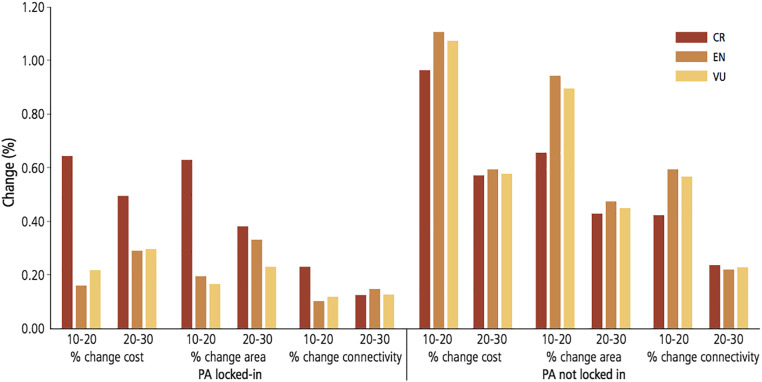
Percentage of change in cost, area, and connectivity from 10 to 20% (10–20) and 20–30% (20–30) of the area of distribution of critically endangered, endangered, and vulnerable raptor species, for the PA locked-in (*Left*) and PA not locked-in (*Right*) strategies (*Materials and Methods*).

The percentages of change of the main parameters (score, cost, number of planning units, connectivity) from the target of 10 to 20% and 20 to 30% were significantly lower in the PA locked-in strategy for all statuses, which indicated that forcing the inclusion of existing protected areas in the solutions implies a lower variability in the models (Fig. 5). However, the transition of a target from 10 to 20% supposed in most cases the highest rise in cost, reserved area, and drop in connectivity than the passage between 20 and 30%, in both strategies (Fig. 5). In other words, a greater effort is required to expand reserved areas from 10 to 20% of species geographic distribution than from 20 to 30%.

**Fig. 6.**
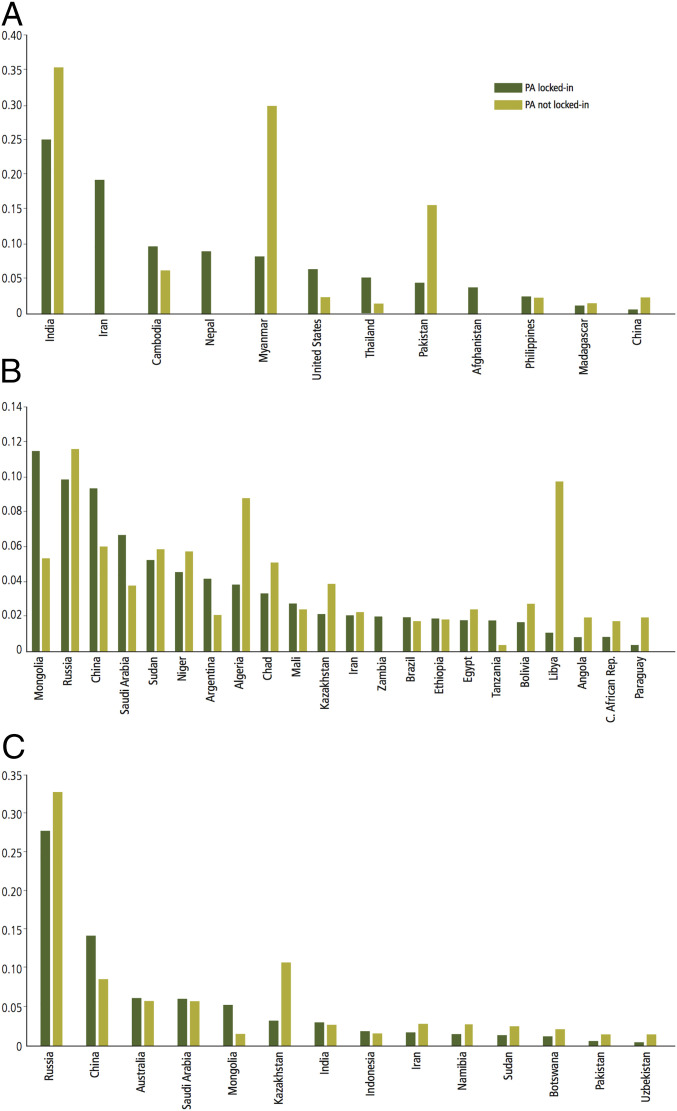
Proportion of the reserve system (target of conservation: 20%) by country for (*A*) critically endangered, (*B*) endangered, and (*C*) vulnerable raptor species. For clarity, only countries with a proportion of the Marxan solutions higher than 2% were included.

In all scenarios, the spatial overlap between the solutions obtained with the two strategies ranged from 6.8% to a maximum of 51.9%; the higher the target (10, 20, or 30%), the broader the overlap (Fig. 5 and Table 2). For critically endangered species, the higher proportion of reserved area selected by both strategies was found in India (Fig. 6*A*). Other countries with substantial proportions of Marxan solutions were located mostly in the southern part of the Asian continent: Iran, Nepal, Cambodia, Myanmar, and Pakistan. Solutions found for endangered species (Fig. 6*B*) encompassed a wider range of countries: Mongolia, Russia, China, Saudi Arabia, Algeria, and Libya were included in the reserve network by both strategies. Russia and China encompassed also large proportions of solutions of both strategies for vulnerable species (Fig. 6*C*) together with Australia, Saudi Arabia, Mongolia, and Kazakhstan.

## Discussion

The tropics have the greatest diversity of raptor species, including hotspots in the Andes and Himalayan and Indo-Malayan regions. These regions have been defined as conservation priority hotspots for all vertebrates (40). Raptor populations are generally affected by human activities such as habitat loss and fragmentation in ecosystems (7, 8, 40–42), and currently about 19% of both diurnal and nocturnal raptors are threatened in the short or long term. Among large predators, raptors are some of the most sensitive taxa to habitat disturbance, stability, and the health of their prey populations because of their complex ecological requirements. Globally, 35 to 40% of the world’s forest area has been converted to other uses, primarily croplands (43, 44). Because of these and other pressures, extinction rates of some vertebrate taxa are 100 times greater than any time in the last 2 million y (4, 6).

Predator mammal and raptor populations are disappearing at a rapid rate (7, 8); one-quarter of vertebrate species are now threatened with extinction and hundreds of thousands of populations have been extirpated in the last century (2, 4). Raptors and other major predators play a major role in ecosystem health, and the removal of such taxa can result in profound changes to ecosystem structure and function, its resilience, and the services it provides (9–11, 45–47). Despite their critical ecological importance, the conservation status of raptors is problematic; globally, 54% of diurnal and 47% of nocturnal species have decreasing populations. Earlier research (46–48) suggested that human population density is the one factor most closely related to the proportion of threatened bird species per nation, but the number of threatened mammal species is more closely related to per-capita gross national product.

### Conservation Prioritization.

We produced reserve system configurations at global levels for each IUCN category of threatened raptor species. Hence, we were able to identify separate hotspots of conservation for critically endangered, endangered, and vulnerable species. Solutions were created for three different targets of area of distribution of species to be protected (10, 20, 30%). Moreover, we compared two strategies of reserve selection: one growing around the existing protected area system and the other based exclusively on target species distribution. The purpose was to evaluate which strategy was more efficient in terms of socioeconomic cost, area, connectivity, and number of species included in the solutions.

The overall small overlap between the reserve networks designed with the two strategies indicated that, inside the range of distribution of threatened raptors, the extant protected area network is located in areas where the socioeconomic cost, and hence the human presence, could assume medium to high values. However, at the spatial resolution of our analysis, the inclusion of current protected areas in the reserve configuration provides the most complete (proportion of conservation targets met) and less variable (changes between models in the same strategy) solutions. Although not directly addressed in this study, it is likely that the monetary and social cost of creating new reserves should easily overcome the differences in the efficiency of the two tested strategies.

Our results indicated that focusing on threatened raptor conservation actions in the existing protected area system could prove to be politically and economically more feasible than creating new reserves, especially in countries where resource allocation in biodiversity conservation is limited. It is important to emphasize, however, that a proper conservation strategy should include consolidating existing reserves first, and then creating new reserves. We found that expanding reserved areas from 10 to 20% of species distribution requires a greater effort (in some cases double) than from 20 to 30%. As might be expected, the greater the proportion of species distribution protected the more efficient their protection became. The highest proportion of the reserve network designed in this study for threatened raptors was located on the Asian and African continents. For critically endangered species, crucial ranges for protected area enhancement or selection were situated in India, Nepal, Iran, Cambodia, and Myanmar. Important areas for conservation of endangered and vulnerable raptors were distributed in Russia, China, Mongolia, and Saudi Arabia. In Africa, threatened raptor conservation should focus on countries such as Sudan, Niger, Algeria, and Namibia.

Our work is one of the first global-scale analyses of the geographic patterns of raptors, a key taxonomic group threatened by habitat modification, and efforts to protect them (7, 8). Our results provide guidelines for global species conservation, focusing both on populations and species extinctions. They show the urgency of solid conservation actions, because of the extremely large number of species showing decreasing populations and being at risk for extinction. Raptors are a good example of the biological annihilation resulting from human activities, the urgency of acting and way of doing it for preventing the extinction crisis, and averting the sixth mass extinction. The fate of all raptors in particular, and biodiversity in general, depends on our conservation actions in the next two decades. Our study is an example of what can be done. But time is running out to save Earth’s biodiversity and avoid a collapse of civilization.

## Materials and Methods

### Spatial Data.

We used BirdLife International database 2015 (https://www.birdlife.org/) and IUCN database 2017 (https://www.iucnredlist.org/) to identify the distribution range for each of the 557 species of raptors. In cases of discrepancies in the spatial data between the two databases the most recent assessment was used or, alternatively, the polygons representing the species range were merged to minimize loss of information. In the original database the GIS (Geographic Information System) polygons were coded based on species presence, origin, and seasonality, and we selected only those where the species were classified as “extant,” where the species origin was “native” or “reintroduced,” and seasonality was classified as “resident,” “breeding season,” “nonbreeding season,” and “passage.” We used the del Hoyo et al. (49) taxonomic guide for species classification and life-history information.

### Global Mapping and Conservation Prioritization.

The study area, which covers the entire global land mass excluding the Antarctic continent, was subdivided into 34,355 100 × 100–km cells. We created maps that represented the following spatial patterns of species richness which we defined as the total number of species in one cell: 1) all species regardless of conservation status or life-history characteristics; 2) diurnal species; 3) nocturnal species; 4) threatened species; 5) species with a national endemism; and 6) species with a restricted range. We defined “threatened” as the species included in the “vulnerable,” “endangered,” and “critically endangered” IUCN categories in contrast to the “least concern” species that include the “least concern” and “near-threatened” categories. National endemism referred to species endemic to a single country and the restricted range species referred to those that have a geographic range equal to or less than 50,000 km^2^ (based on the program criteria for the Important Bird and Biodiversity Area program of Birdlife International; http://www.birdlife.org//worldwide/science).

We used ArcMap 10.1 (50) for the mapping and spatial analysis and Marxan (v2.4.3), an algorithm designed to identify the reserve system that meets specific biodiversity conservation targets at the minimum cost, for the conservation planning analysis (51, 52).

To simulate the reserve system through Marxan, we set three different targets of areas to be protected based on the status of a species: 10, 20, and 30% of the area of the range distribution for critically endangered (CR), endangered (EN), vulnerable (VU), and nonthreatened species (near-threatened [NT] and least concern [LC] categories of the IUCN classification). In order to minimize the conflict between conservation targets and human activities, we incorporated into the Marxan model an index of relative cost to lands with different degrees of naturalness and human impact. Specifically, the index of relative cost was based on three land-cover types: natural ecosystems, rainfed cropland, and irrigated cropland and urbanized areas. We made three assumptions underlying the assignment of the relative cost of each land-cover type: 1) Natural ecosystems are more suitable for the conservation of most raptor species than areas of human activity in ecological, economic, and social terms and hence they have a lower relative cost; 2) rainfed croplands, which have intermediate relative costs, are less suitable than natural ecosystems but more suitable than irrigated croplands and urbanized areas because they require a lower input of energy and have less pollution: and 3) irrigated croplands and urbanized areas have the highest relative cost for inclusion into a reserve network because they are considered the least suitable environments for raptor conservation.

We compiled the “natural ecosystems” layer in ArcMap by combining land-cover categories extracted from Global Land Cover (GLC) 2012; the categories included forest, shrubland, savanna, grassland, wetland, snow and ice, and barren or sparsely vegetated areas (53). We used the Global Agro-Ecological Zones Database (GAEZ; v3.0) (54) to obtain the spatial data on rainfed croplands, and we combined data from GAEZ (v3.0) and the urban and urbanized category of GLC 2012 to produce the irrigated cropland and urbanized area layer. For each cell, we calculated the percentage of each land-cover type and assigned a relative value to each of the four class ranges of cover: 0 to 25%, 26 to 50%, 51 to 75%, and 76 to 100%. Following the above assumptions, the higher the percentage of natural ecosystem cover the lower the cost and, conversely, the higher the percentage of cover of rainfed cropland and irrigated cropland and urbanized areas the higher the cost.

Moreover, to test how close and how efficiently the existing PA system achieves the stated ecological objectives, we explored two strategies of reserve system planning: 1) one in which the Marxan algorithm was forced to include in the final solution the existing protected area system (PA locked-in scenario); and 2) one in which the algorithm had no constraints in meeting the stated conservation targets, except minimizing the socioeconomic cost (PA not locked-in scenario).

A planning unit (or grid cell of the study area) was classified as belonging to the existing PA system if more than 25% of its surface was occupied by areas reported in the World Database on Protected Areas (55) and was part of one of the IUCN categories (Ia, strict nature reserve; Ib, wilderness area; II, national park; III, natural monument or feature; IV, habitat/species management area; V, protected landscape; VI, protected area with sustainable use of natural resources). The 24 best solutions for each model (two strategies, three different targets of area, five conservation categories) were compared using the following parameters: 1) efficiency: score, cost, and number of planning units of the Marxan solution; 2) connectivity: sum of the boundary length of the contiguous site; 3) completeness: number of species not meeting the conservation targets; 4) overlay: proportion of planning units selected by the same models (same target of area and same conservation category) in each strategy; 5) proportion of planning units in the solution that overlap with the existing PA system; and 6) percentage of change in cost, area, and connectivity from 10 to 20% and from 20 to 30% of species distribution to be protected.

Marxan’s objective function parameters (i.e., boundary length modifier, species penalty factor) were set to the same value for both strategies after calibration. The number of model iterations was set to 1,000, and the final solution was the average of the iterations. For each pair of solutions compared (same target and conservation status, different strategy), a cost threshold was set to the lower cost value achieved, meaning that the reserve networks were forced not to exceed the same cost value. However, it should be noted that the Marxan algorithm (simulated annealing) allows the system to go above the cost threshold in order to find a reasonably efficient solution. Finally, the solutions were mapped and their spatial distribution by country was analyzed in ArcMap 10.1.

## Supplementary Material

Supplementary File

## Data Availability

All study data are included in the article and/or *SI Appendix*.

## Change History

September 6, 2021: The article text has been updated.
