# Supplementary material for: Global patterns of raptor distribution and protected areas optimal selection to reduce the extinction crises
Source: Proc Natl Acad Sci U S A. 2021 Aug 30;118(37):e2018203118. doi: 10.1073/pnas.2018203118 (PMC8449406; doi:10.1073/pnas.2018203118)
Supplement: Supplementary File [file pnas.2018203118.sapp.pdf]

**Cruz et al.**

**Supplementary Materials**

**Summary of information on raptor species diversity by country.**

| Continent | Country Name | All species (n) | Diurnal Species (n) | Nocturnal Species (n) | Threatened Species (n) | Endemisms (n) | Restricted Distribution (n) |
|---|---|---|---|---|---|---|---|
| Africa | Algeria | 40 | 32 | 8 | 3 | 0 | 0 |
| | Angola | 80 | 65 | 15 | 7 | 0 | 0 |
| | Benin | 66 | 54 | 12 | 9 | 0 | 0 |
| | Botswana | 67 | 56 | 11 | 8 | 0 | 0 |
| | Burkina Faso | 60 | 50 | 10 | 9 | 0 | 0 |
| | Burundi | 64 | 51 | 13 | 9 | 1 | 2 |
| | Cameroon | 77 | 57 | 20 | 9 | 0 | 0 |
| | Cape Verde | 7 | 5 | 2 | 0 | 0 | 0 |
| | Central African Republic | 77 | 59 | 18 | 9 | 0 | 0 |
| | Chad | 63 | 51 | 12 | 10 | 0 | 0 |
| | Comoros | 8 | 4 | 4 | 3 | 3 | 2 |
| | Congo | 53 | 35 | 18 | 0 | 0 | 0 |
| | Congo, DRC | 96 | 69 | 27 | 9 | 1 | 2 |
| | Cote d'Ivoire | 73 | 55 | 18 | 9 | 0 | 0 |
| | Djibouti | 43 | 37 | 6 | 9 | 0 | 0 |
| | Egypt | 38 | 29 | 9 | 5 | 0 | 0 |
| | Equatorial Guinea | 34 | 23 | 11 | 0 | 0 | 0 |
| | Eritrea | 70 | 57 | 13 | 11 | 0 | 0 |
| | Ethiopia | 85 | 70 | 15 | 12 | 0 | 0 |
| | Gabon | 45 | 29 | 16 | 0 | 0 | 0 |
| | Gaza Strip | 25 | 18 | 7 | 4 | 0 | 0 |
| | Ghana | 72 | 55 | 17 | 9 | 0 | 0 |
| | Guinea | 69 | 53 | 16 | 8 | 0 | 0 |
| | Guinea-Bissau | 53 | 44 | 9 | 8 | 0 | 0 |
| | Israel | 37 | 26 | 11 | 4 | 0 | 0 |
| | Kenya | 90 | 71 | 19 | 13 | 0 | 1 |
| | Lesotho | 37 | 30 | 7 | 5 | 0 | 0 |
| | Liberia | 54 | 38 | 16 | 4 | 0 | 0 |
| | Libya | 21 | 15 | 6 | 2 | 0 | 0 |
| | Madagascar | 23 | 16 | 7 | 5 | 13 | 2 |
| | Malawi | 68 | 55 | 13 | 7 | 0 | 0 |
| | Mali | 64 | 51 | 13 | 10 | 0 | 0 |
| | Mauritania | 57 | 46 | 11 | 10 | 0 | 0 |

| | | | | | | | |
|---|---|---|---|---|---|---|---|
| | Mauritius | 1 | 1 | 0 | 1 | 1 | 1 |
| | Mayotte | 6 | 3 | 3 | 0 | 1 | 1 |
| | Morocco | 37 | 28 | 9 | 1 | 0 | 0 |
| | Mozambique | 70 | 57 | 13 | 8 | 0 | 0 |
| | Namibia | 68 | 56 | 12 | 9 | 0 | 0 |
| | Niger | 64 | 52 | 12 | 10 | 0 | 0 |
| | Nigeria | 74 | 58 | 16 | 9 | 0 | 0 |
| | Portugal | 32 | 24 | 8 | 2 | 0 | 0 |
| | Reunion | 1 | 1 | 0 | 1 | 1 | 1 |
| | Rwanda | 70 | 54 | 16 | 9 | 1 | 2 |
| | Sao Tome & Principe | 5 | 2 | 3 | 1 | 1 | 1 |
| | Senegal | 65 | 53 | 12 | 9 | 0 | 0 |
| | Seychelles | 6 | 3 | 3 | 2 | 3 | 2 |
| | Sierra Leone | 56 | 42 | 14 | 4 | 0 | 0 |
| | Somalia | 64 | 52 | 12 | 9 | 0 | 0 |
| | South Africa | 79 | 66 | 13 | 10 | 1 | 0 |
| | Spain | 38 | 28 | 10 | 2 | 0 | 0 |
| | Sudan | 87 | 73 | 14 | 13 | 0 | 0 |
| | Swaziland | 61 | 48 | 13 | 7 | 0 | 0 |
| | Tanzania | 83 | 67 | 16 | 13 | 1 | 2 |
| | The Gambia | 60 | 49 | 11 | 8 | 0 | 0 |
| | Togo | 62 | 53 | 9 | 8 | 0 | 0 |
| | Tunisia | 35 | 27 | 8 | 2 | 0 | 0 |
| | Uganda | 90 | 71 | 19 | 9 | 0 | 0 |
| | Western Sahara | 18 | 13 | 5 | 3 | 0 | 0 |
| | Zambia | 69 | 57 | 12 | 7 | 0 | 0 |
| | Zimbabwe | 71 | 58 | 13 | 8 | 0 | 0 |
| **Asia** | Afghanistan | 45 | 34 | 11 | 6 | 0 | 0 |
| | Armenia | 39 | 31 | 8 | 4 | 0 | 0 |
| | Azerbaijan | 39 | 31 | 8 | 4 | 0 | 0 |
| | Bahrain | 10 | 7 | 3 | 1 | 0 | 0 |
| | Bangladesh | 66 | 48 | 18 | 8 | 1 | 0 |
| | Bhutan | 66 | 46 | 20 | 5 | 1 | 0 |
| | Brunei | 33 | 24 | 9 | 2 | 2 | 1 |
| | Bulgaria | 44 | 33 | 11 | 4 | 0 | 0 |
| | Cambodia | 52 | 37 | 15 | 6 | 1 | 0 |
| | China | 93 | 64 | 29 | 10 | 3 | 0 |
| | Cyprus | 23 | 17 | 6 | 2 | 0 | 0 |
| | Georgia | 41 | 33 | 8 | 4 | 0 | 0 |
| | Greece | 44 | 34 | 10 | 4 | 0 | 0 |
| | India | 101 | 67 | 34 | 12 | 10 | 8 |
| | Indonesia | 116 | 64 | 52 | 14 | 43 | 29 |

| | | | | | | | |
|---|---|---|---|---|---|---|---|
| | Iran | 51 | 40 | 11 | 6 | 0 | 0 |
| | Iraq | 36 | 28 | 8 | 4 | 0 | 0 |
| | Japan | 32 | 22 | 10 | 4 | 2 | 1 |
| | Jordan | 37 | 26 | 11 | 4 | 0 | 0 |
| | Kazakhstan | 47 | 35 | 12 | 5 | 0 | 0 |
| | Kuwait | 21 | 15 | 6 | 4 | 0 | 0 |
| | Kyrgyzstan | 39 | 30 | 9 | 5 | 0 | 0 |
| | Laos | 59 | 43 | 16 | 5 | 1 | 0 |
| | Lebanon | 28 | 21 | 7 | 4 | 0 | 0 |
| | Malaysia | 53 | 35 | 18 | 5 | 4 | 2 |
| | Mongolia | 46 | 35 | 11 | 4 | 0 | 0 |
| | Myanmar | 83 | 60 | 23 | 9 | 1 | 0 |
| | Nepal | 80 | 59 | 21 | 9 | 1 | 0 |
| | North Korea | 33 | 22 | 11 | 4 | 2 | 0 |
| | Oman | 27 | 18 | 9 | 5 | 0 | 1 |
| | Pakistan | 68 | 50 | 18 | 8 | 0 | 0 |
| | Papua New Guinea | 52 | 36 | 16 | 11 | 12 | 13 |
| | Philippines | 51 | 26 | 25 | 11 | 24 | 12 |
| | Qatar | 13 | 10 | 3 | 3 | 0 | 0 |
| | Russia | 63 | 46 | 17 | 8 | 2 | 0 |
| | Saudi Arabia | 40 | 32 | 8 | 5 | 0 | 0 |
| | Singapore | 26 | 18 | 8 | 0 | 1 | 0 |
| | South Korea | 29 | 19 | 10 | 3 | 2 | 0 |
| | Sri Lanka | 38 | 25 | 13 | 1 | 3 | 2 |
| | Syria | 36 | 25 | 11 | 4 | 0 | 0 |
| | Tajikistan | 38 | 30 | 8 | 5 | 0 | 0 |
| | Thailand | 68 | 46 | 22 | 6 | 1 | 0 |
| | Timor Leste | 17 | 14 | 3 | 0 | 0 | 0 |
| | Turkey | 46 | 35 | 11 | 4 | 0 | 0 |
| | Turkmenistan | 37 | 29 | 8 | 5 | 0 | 0 |
| | United Arab Emirates | 19 | 14 | 5 | 5 | 0 | 0 |
| | Uzbekistan | 37 | 29 | 8 | 5 | 0 | 0 |
| | Vietnam | 62 | 44 | 18 | 5 | 1 | 0 |
| | West Bank | 32 | 24 | 8 | 4 | 0 | 0 |
| | Yemen | 34 | 27 | 7 | 5 | 2 | 2 |
| **Australia** | Australia | 35 | 24 | 11 | 1 | 10 | 0 |
| **Europe** | Albania | 37 | 29 | 8 | 2 | 0 | 0 |
| | Andorra | 20 | 14 | 6 | 1 | 0 | 0 |
| | Austria | 36 | 25 | 11 | 3 | 0 | 0 |
| | Belarus | 38 | 25 | 13 | 3 | 0 | 0 |
| | Belgium | 22 | 14 | 8 | 0 | 0 | 0 |

| | | | | | | | |
|---|---|---|---|---|---|---|---|
| | Bosnia & Herzegovina | 39 | 29 | 10 | 4 | 0 | 0 |
| | Croatia | 40 | 29 | 11 | 3 | 0 | 0 |
| | Czech Republic | 30 | 20 | 10 | 2 | 0 | 0 |
| | Denmark | 24 | 16 | 8 | 0 | 0 | 0 |
| | Estonia | 32 | 20 | 12 | 1 | 0 | 0 |
| | Faroe Is. | 1 | 1 | 0 | 0 | 0 | 0 |
| | Finland | 27 | 17 | 10 | 1 | 0 | 0 |
| | France | 38 | 28 | 10 | 2 | 0 | 0 |
| | Germany | 30 | 19 | 11 | 0 | 0 | 0 |
| | Guernsey | 4 | 1 | 3 | 0 | 0 | 0 |
| | Hungary | 35 | 26 | 9 | 3 | 0 | 0 |
| | Iceland | 6 | 3 | 3 | 0 | 0 | 0 |
| | Ireland | 10 | 6 | 4 | 0 | 0 | 0 |
| | Isle of Man | 8 | 4 | 4 | 0 | 0 | 0 |
| | Italy | 40 | 30 | 10 | 3 | 0 | 0 |
| | Jersey | 4 | 1 | 3 | 0 | 0 | 0 |
| | Latvia | 33 | 20 | 13 | 1 | 0 | 0 |
| | Liechtenstein | 19 | 12 | 7 | 0 | 0 | 0 |
| | Lithuania | 33 | 20 | 13 | 1 | 0 | 0 |
| | Luxembourg | 22 | 14 | 8 | 0 | 0 | 0 |
| | Macedonia | 38 | 30 | 8 | 4 | 0 | 0 |
| | Malta | 4 | 4 | 0 | 1 | 0 | 0 |
| | Moldova | 35 | 27 | 8 | 4 | 0 | 0 |
| | Netherlands | 22 | 15 | 7 | 0 | 0 | 0 |
| | Norway | 24 | 14 | 10 | 0 | 0 | 0 |
| | Poland | 36 | 24 | 12 | 2 | 0 | 0 |
| | Romania | 40 | 29 | 11 | 4 | 0 | 0 |
| | San Marino | 16 | 10 | 6 | 0 | 0 | 0 |
| | Serbia & Montenegro | 41 | 31 | 10 | 4 | 0 | 0 |
| | Slovakia | 36 | 25 | 11 | 3 | 0 | 0 |
| | Slovenia | 33 | 22 | 11 | 1 | 0 | 0 |
| | Sweden | 29 | 18 | 11 | 0 | 0 | 0 |
| | Switzerland | 28 | 19 | 9 | 0 | 0 | 0 |
| | Ukraine | 42 | 30 | 12 | 4 | 0 | 0 |
| | United Kingdom | 22 | 16 | 6 | 0 | 0 | 0 |
| **North America** | Anguilla | 7 | 7 | 0 | 0 | 0 | 0 |
| | Antigua & Barbuda | 10 | 8 | 2 | 0 | 0 | 0 |
| | Aruba | 4 | 2 | 2 | 0 | 0 | 0 |
| | Barbados | 7 | 7 | 0 | 0 | 0 | 0 |
| | Belize | 57 | 44 | 13 | 0 | 0 | 0 |
| | Canada | 39 | 22 | 17 | 0 | 1 | 0 |
| | Cayman Is. | 10 | 7 | 3 | 0 | 0 | 0 |

| | | | | | | | |
|---|---|---|---|---|---|---|---|
| | Colombia | 103 | 76 | 27 | 4 | 0 | 6 |
| | Costa Rica | 71 | 54 | 17 | 0 | 0 | 3 |
| | Cuba | 24 | 17 | 7 | 2 | 5 | 3 |
| | Dominica | 10 | 8 | 2 | 0 | 0 | 0 |
| | Dominican Republic | 16 | 11 | 5 | 1 | 1 | 1 |
| | El Salvador | 49 | 34 | 15 | 0 | 0 | 1 |
| | Greenland | 4 | 2 | 2 | 0 | 0 | 0 |
| | Grenada | 11 | 9 | 2 | 0 | 0 | 0 |
| | Guadeloupe | 9 | 7 | 2 | 0 | 0 | 0 |
| | Guatemala | 70 | 49 | 21 | 1 | 0 | 2 |
| | Haiti | 15 | 10 | 5 | 0 | 0 | 0 |
| | Honduras | 67 | 50 | 17 | 0 | 0 | 0 |
| | Jamaica | 14 | 11 | 3 | 0 | 1 | 1 |
| | Martinique | 8 | 8 | 0 | 0 | 0 | 0 |
| | Mexico | 91 | 57 | 34 | 2 | 5 | 3 |
| | Montserrat | 6 | 6 | 0 | 0 | 0 | 0 |
| | Netherlands Antilles | 5 | 2 | 3 | 0 | 0 | 0 |
| | Nicaragua | 67 | 52 | 15 | 0 | 0 | 0 |
| | Panama | 73 | 57 | 16 | 1 | 0 | 3 |
| | Puerto Rico | 15 | 11 | 4 | 0 | 1 | 1 |
| | St. Kitts & Nevis | 9 | 7 | 2 | 0 | 0 | 0 |
| | St. Lucia | 8 | 8 | 0 | 0 | 0 | 0 |
| | St. Pierre & Miquelon | 6 | 4 | 2 | 0 | 0 | 0 |
| | St. Vincent & the Grenadines | 11 | 9 | 2 | 0 | 0 | 0 |
| | The Bahamas | 12 | 10 | 2 | 0 | 0 | 0 |
| | Trinidad & Tobago | 41 | 34 | 7 | 0 | 0 | 0 |
| | Turks & Caicos Is. | 10 | 8 | 2 | 0 | 0 | 0 |
| | United States | 61 | 37 | 24 | 1 | 5 | 2 |
| | Virgin Is. | 12 | 9 | 3 | 0 | 1 | 1 |
| **Oceania** | American Samoa | 2 | 0 | 2 | 0 | 0 | 0 |
| | Chile | 31 | 23 | 8 | 1 | 0 | 0 |
| | Fiji | 6 | 3 | 3 | 0 | 1 | 1 |
| | French Polynesia | 1 | 1 | 0 | 0 | 0 | 0 |
| | Guam | 2 | 2 | 0 | 0 | 0 | 0 |
| | New Caledonia | 10 | 7 | 3 | 0 | 1 | 1 |
| | New Zealand | 6 | 3 | 3 | 0 | 1 | 0 |
| | Niue | 2 | 0 | 2 | 0 | 0 | 0 |
| | Palau | 3 | 1 | 2 | 0 | 1 | 1 |
| | Samoa | 2 | 0 | 2 | 0 | 0 | 0 |
| | Solomon Is. | 19 | 12 | 7 | 3 | 2 | 8 |
| | Tonga | 2 | 0 | 2 | 0 | 0 | 0 |
| | Vanuatu | 5 | 3 | 2 | 0 | 0 | 0 |

| | | | | | | | |
|---|---|---|---|---|---|---|---|
| South America | Argentina | 86 | 62 | 24 | 2 | 0 | 0 |
| | Bolivia | 96 | 70 | 26 | 2 | 0 | 1 |
| | Brazil | 95 | 73 | 22 | 3 | 2 | 1 |
| | Ecuador | 102 | 73 | 29 | 6 | 1 | 8 |
| | Falkland Is. | 12 | 7 | 5 | 0 | 0 | 0 |
| | French Guiana | 68 | 54 | 14 | 0 | 0 | 0 |
| | Guyana | 72 | 55 | 17 | 0 | 0 | 0 |
| | Paraguay | 75 | 55 | 20 | 1 | 0 | 0 |
| | Peru | 102 | 70 | 32 | 4 | 2 | 6 |
| | Suriname | 67 | 53 | 14 | 0 | 0 | 0 |
| | Uruguay | 40 | 30 | 10 | 1 | 0 | 0 |
| | Venezuela | 88 | 66 | 22 | 1 | 0 | 0 |
